# Supplementary material for: Prrx1-driven LINC complex disruption in vivo reduces osteoid deposition but not bone quality after voluntary wheel running
Source: PLoS One. 2024 Nov 20;19(11):e0307816. doi: 10.1371/journal.pone.0307816 (PMC11578491; doi:10.1371/journal.pone.0307816)
Supplement: S1 File — (PDF) [file pone.0307816.s001.pdf]

# *Supplementary Figures*

## ***Prrx1-driven LINC complex disruption in vivo reduces osteoid deposition but not bone quality after voluntary wheel running***

Scott Birks[1], Sean Howard[2], Christian S. Wright[3], Caroline O'Rourke[4], Elicza A. Day[3], Alexander J. Lamb[3], James R. Walsdorf[3], Anthony Lau[4], William R. Thompson[3], Gunes Uzer[2]†

[1]Boise State University, Micron School of Materials Science and Engineering
[2]Boise State University, Mechanical and Biomedical Engineering
[3]Indiana University, Department of Physical Therapy, School of Health and Human Sciences
[4]The College of New Jersey, Biomedical Engineering

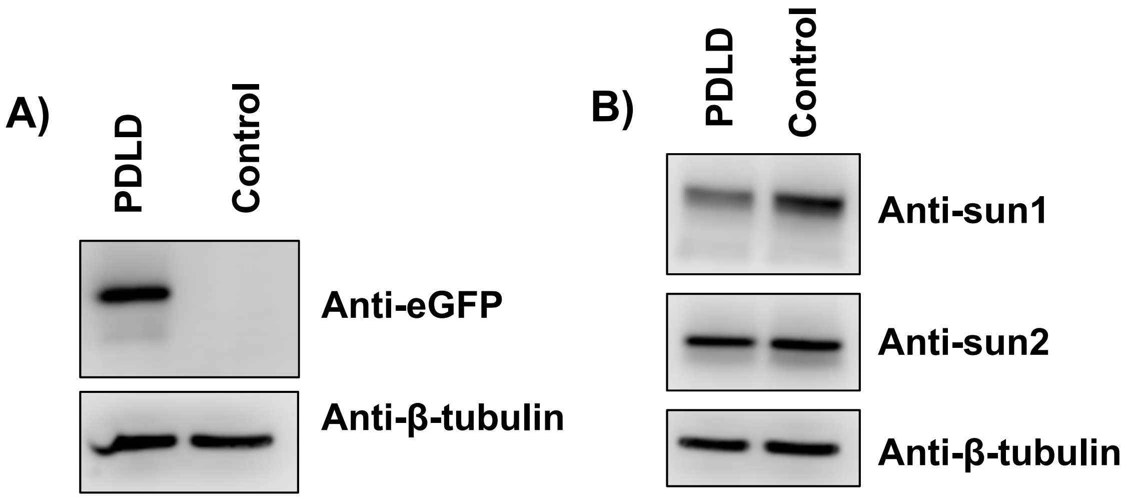


**Figure S1** Western blot against eGFP showing **(A)** the presence of eGFP in bone marrow cells of PDLD mice and not in control mice. Anti-B tublin was used as a positive control. **(B)** indicates decreased levels of sun1 and sun2 in PDLD mice.

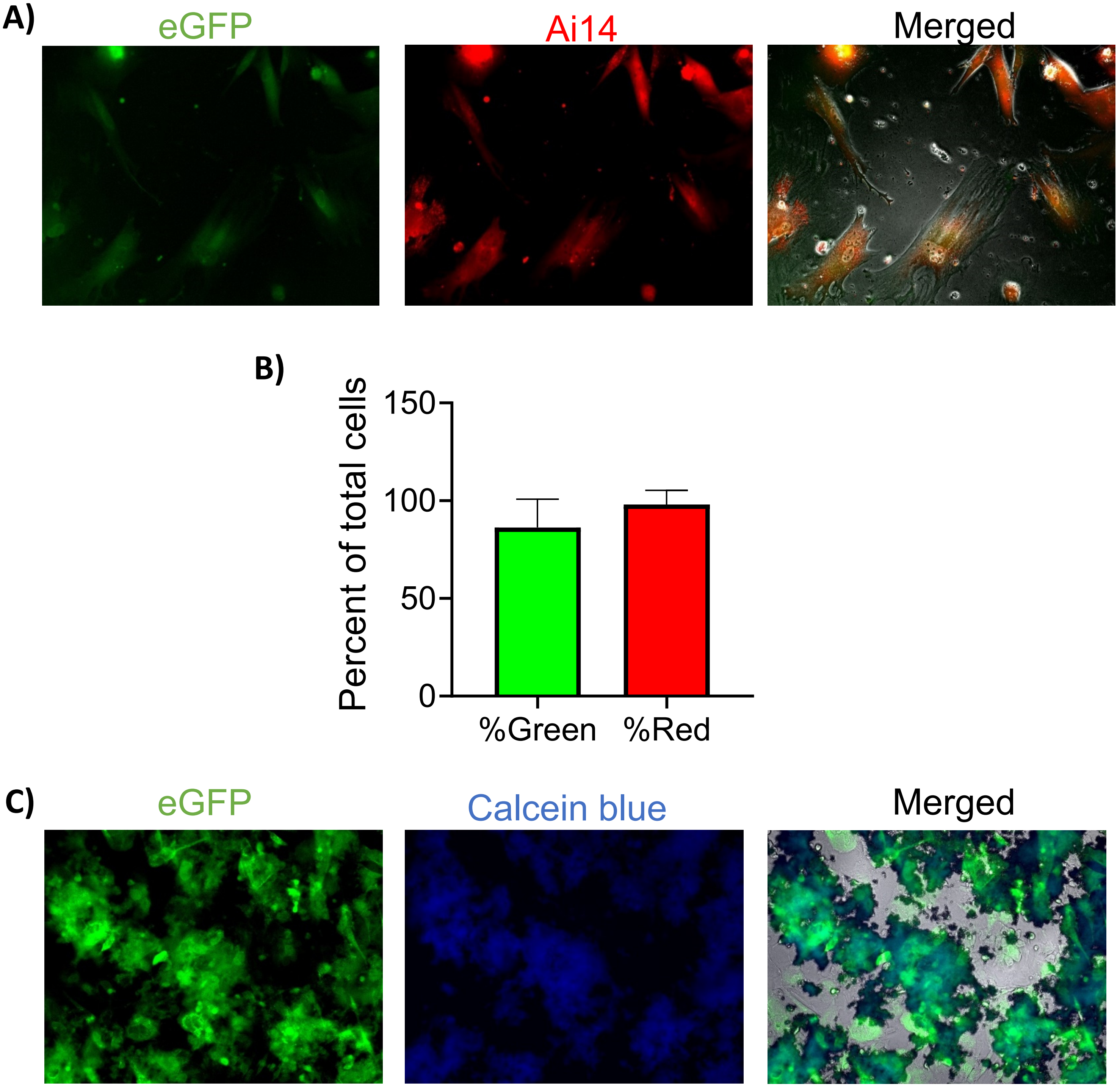


**Figure S2 eGFP-KASH expressing cells in primary cell culture. A)** Representative images of bone marrow aspirated from 12-week female Prx-Cre(+)/Ai14(+)/KASH(+) Number of cells per field expressing the GFP-KASH construct was determined by fluorescent microscopy in the control cells of Prx lineage were determined by tdTomato expression in both the control All counts were done utilizing FIJI. **B)** Graph of each cell percentages to total, error bars represent standard deviation. **C) KASH-GFP expressing cells contribute to the formation of osteogenic nodules.** Images were taken at 10X using a) FITC filter showing eGFP expression b), DAPI filter showing calcein blue fluorescence c) an overlay to qualitatively show that the green KASH-GFP expressing cells were located in the blue Calcein Blue stained nodule region

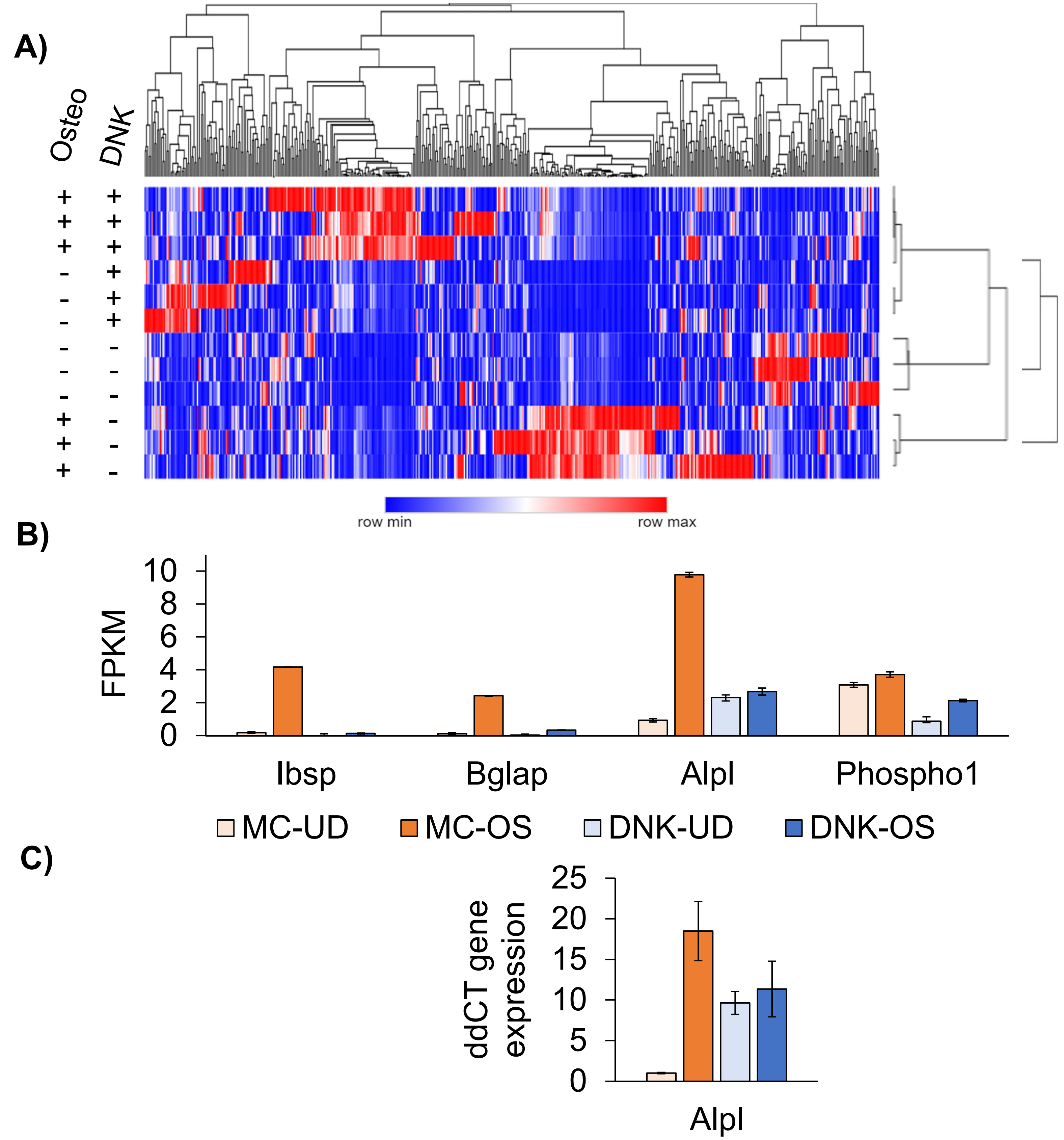


**Figure S3. RNA-seq Analysis of DNK and MC Expressing MSCs During Osteogenesis**: (**A**) Heatmap of FPKM values for mCherry tagged dominant negative KASH (DNK) and empty mCherry (MC) plasmids expressing MSCs during 7 days of osteogenesis all shown values were p<0.05. (**B**) FPKM values of osteogenesis genes of Ibsp, Bglap, Alpl, and Phospho1 in MSCs expressing DNK or MC plasmid during 7-day osteogenesis (**C**) qPCR quantification of same samples using Alpl primer (Forward: aacccagacacaagcattcc, Reverse: gcctttgaggtttttggtca). Both undifferentiated controls (UD) and osteogenic media (OS) was shown.

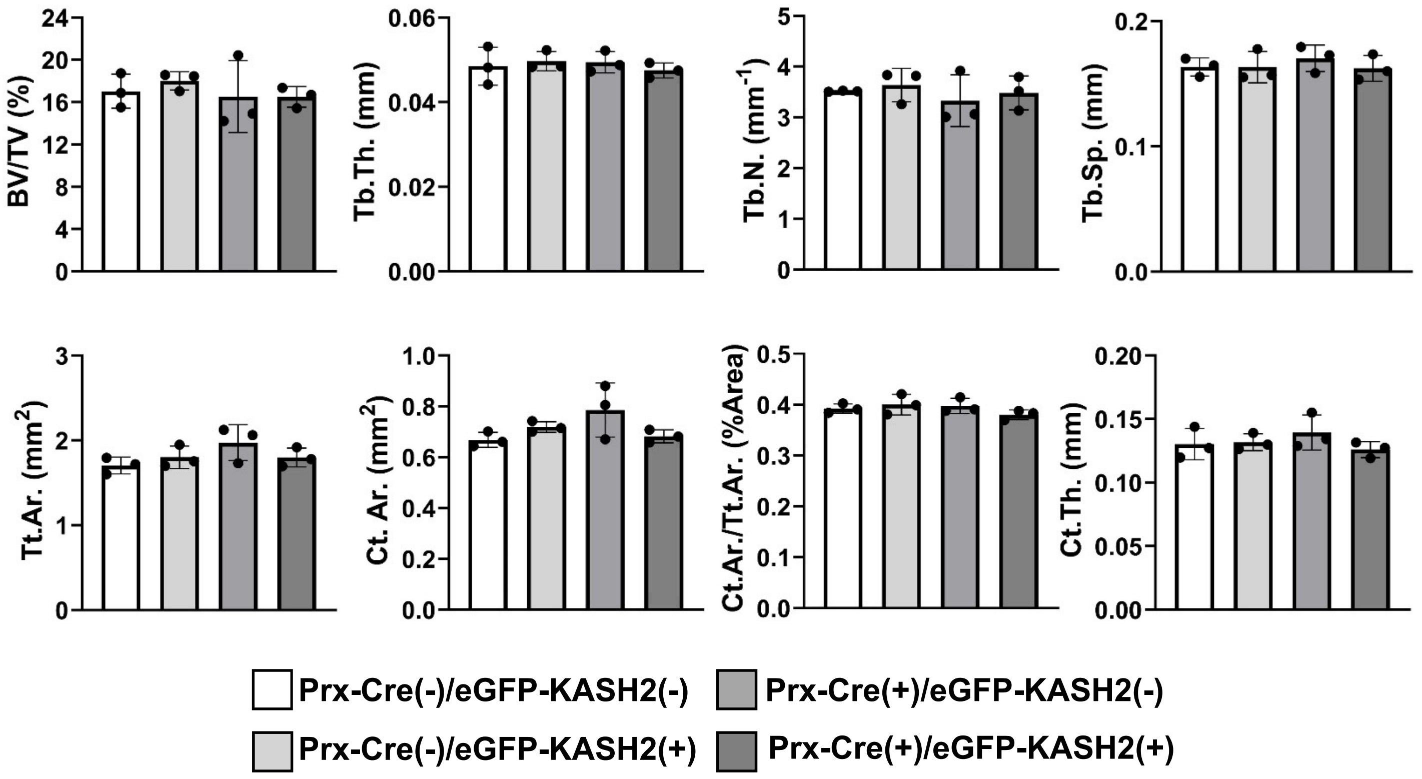


**Figure S4. Bone microarchitecture for all four possible genotypes at 8-weeks** (n=3/grp): Prx-Cre(+)/EGFP-KASH2(+), Prx-Cre(+)/EGFP-KASH2(-), Prx-Cre(-)/EGFP-KASH2(+), Prx-Cre(-)/EGFP-KASH2(-). There was no significant difference found between genotypes. Significance determined via ordinary one-way ANOVA.

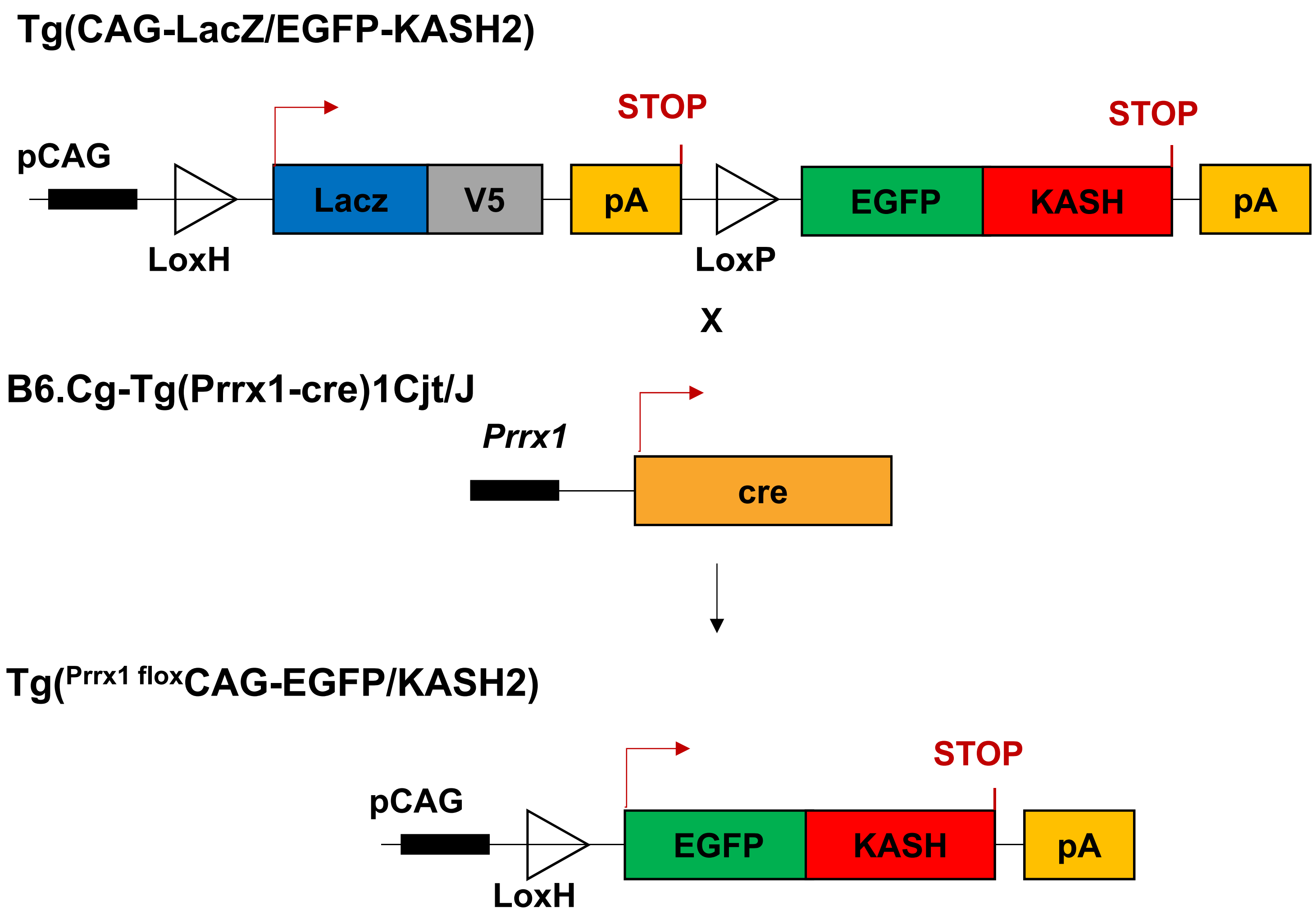


**Figure S5. Breeding scheme for the creation of PDLD mice.** Hemizygous Prx-Cre mice were crossed with floxed KASH2 mice to generate Prrx1-driven LINC disrupted (PDLD) murine model. LINC disruption mechanism described by Razafsky and Hodzic**.** Genotyping was performed on tissue biopsies via real-time PCR probing for LacZ and Cre (Transnetyx, Cordova, TN) to determine experimental animals and controls. Cre(+)/LacZ(+) animals were considered experimental and all other genotypes were considered controls.

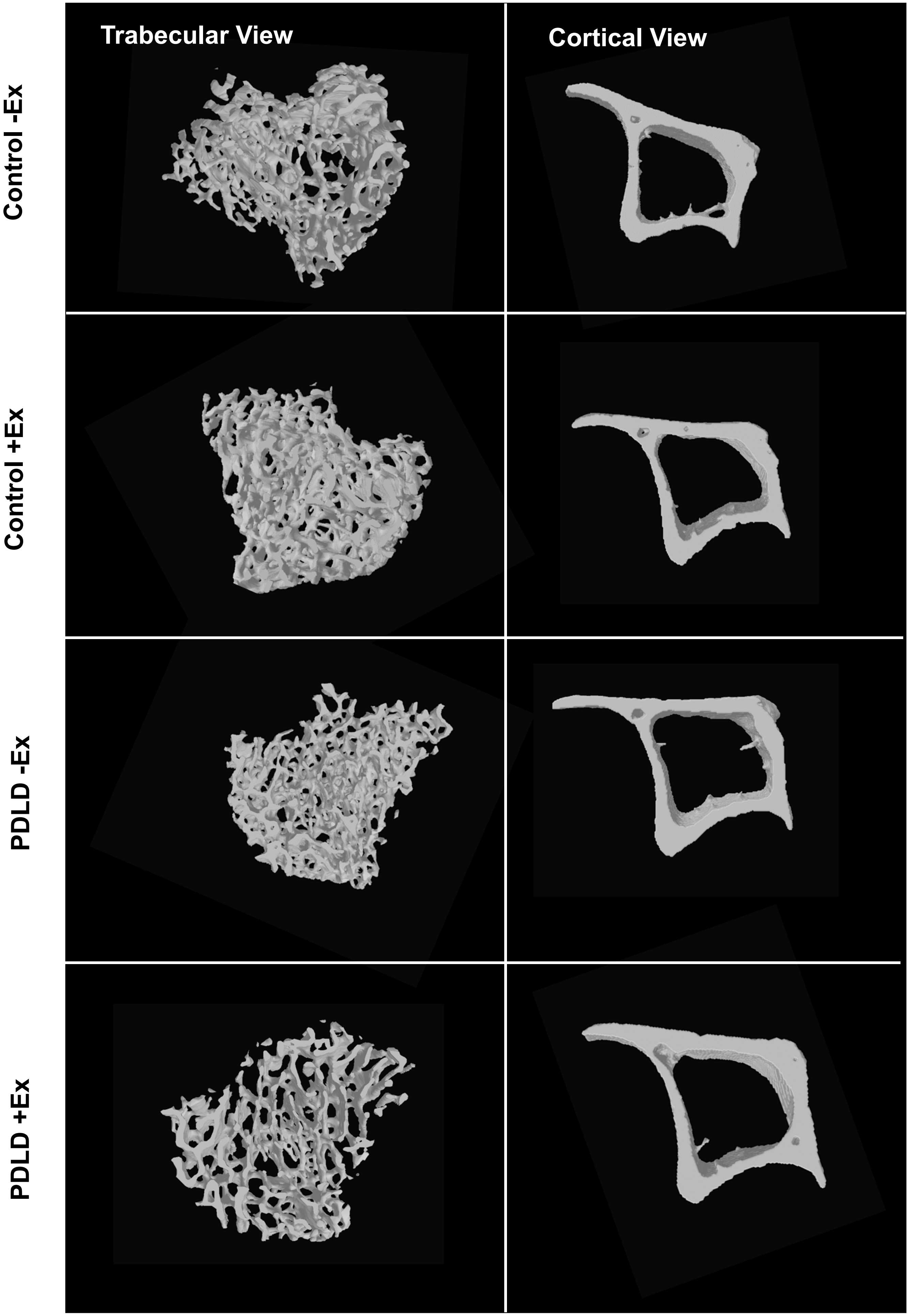


**Figure S6.** Representative micro-CT images for trabecular and cortical architecture of control and PDLD animals with and without exercise.
