# Supplementary material for: Prrx1-driven LINC complex disruption in vivo reduces osteoid deposition but not bone quality after voluntary wheel running
Source: PLoS One. 2024 Nov 20;19(11):e0307816. doi: 10.1371/journal.pone.0307816 (PMC11578491; doi:10.1371/journal.pone.0307816)
Supplement: S2 File — (DOCX) [file pone.0307816.s002.docx]

**Table S1.** GO Term DAVID analysis of genes down regulated in MC vs KASH expressing MSCs. All presented genes were p<0.05 between treatment groups and had a Log Fold Change <1

| Term | Benjamini | Genes | Fold Enrichment |
| --- | --- | --- | --- |
| GO:0046069~cGMP catabolic process | 2.7E-02 | PDE10A, PDE1A, PDE5A, PDE9A | 30 |
| GO:0072534~perineuronal net | 2.3E-03 | BCAN, ACAN, PTPRZ1, HAPLN4, HAPLN1 | 19 |
| GO:0004114~3',5'-cyclic-nucleotide phosphodiesterase activity | 1.9E-03 | PDE10A, PDE1B, PDE1A, PDE3B, PDE5A, PDE8A, PDE9A | 12 |
| GO:0004115~3',5'-cyclic-AMP phosphodiesterase activity | 1.9E-03 | PDE10A, PDE1B, PDE1A, PDE3B, PDE5A, PDE8A, PDE9A | 12 |
| GO:0047555~3',5'-cyclic-GMP phosphodiesterase activity | 2.9E-03 | PDE10A, PDE1B, PDE1A, PDE3B, PDE5A, PDE8A, PDE9A | 11 |
| GO:0030020~extracellular matrix structural constituent conferring tensile strength | 1.2E-04 | COL1A1, COL2A1, COL14A1, COL4A4, COL11A1, COL11A2, COL9A1, COL4A6, COL8A1, COL9A3, COL9A2 | 9 |
| GO:0030020~extracellular matrix structural constituent conferring tensile strength | 1.2E-04 | COL1A1, COL2A1, COL14A1, COL4A4, COL11A1, COL11A2, COL9A1, COL4A6, COL8A1, COL9A3, COL9A2 | 9 |
| GO:0005540~hyaluronic acid binding | 2.9E-02 | BCAN, ACAN, CEMIP, SUSD5, HAPLN4, HAPLN1 | 9 |
| GO:0005540~hyaluronic acid binding | 2.9E-02 | BCAN, ACAN, CEMIP, SUSD5, HAPLN4, HAPLN1 | 9 |
| GO:0043083~synaptic cleft | 1.4E-02 | DNM3, LAMA5, LAMA2, LAMA4, SLC1A1, GRIN1 | 8 |
| GO:0015459~potassium channel regulator activity | 4.1E-03 | KCNS1, KCNE4, AMIGO1, KCNMB4, SGK3, ANK2, KCNAB2, ADRB2 | 8 |
| GO:0001975~response to amphetamine | 1.2E-02 | PDE1B, PPP1R1B, SLC1A1, RGS10, ADRA1B, HDAC9, SLC18A2, GRIN1 | 8 |
| GO:0008081~phosphoric diester hydrolase activity | 1.3E-04 | SMPD3, SMPD5, PDE10A, PDE1B, PLCL1, PDE1A, PDE3B, PLCE1, PDE5A, SMPDL3A, PDE8A, PDE9A | 7 |
| GO:0030199~collagen fibril organization | 8.5E-04 | GREM1, COL1A1, MMP11, ACAN, COL2A1, COL14A1, COL11A1, COL11A2, SCX, DPT, FMOD | 7 |
| GO:0005581~collagen trimer | 5.1E-05 | CCBE1, EDA, COL14A1, COL11A1, COL11A2, C1QL4, COL1A1, C1QTNF3, COL2A1, COL4A4, COL9A1, COL8A1, COL9A2 | 6 |
| GO:0005604~basement membrane | 6.5E-05 | LAMA5, LAMA2, LAMA4, LAMA3, NTN4, PTN, THBS2, NPNT, SMOC2, ACAN, COL2A1, COL4A4, COL4A6, TIMP3, COL8A1 | 5 |
| GO:0007160~cell-matrix adhesion | 9.5E-03 | BCAN, EDA, MIA, TNN, ITGA10, ITGA2, ITGA8, ITGB8, ITGBL1, SIRPA, L1CAM, NPNT | 5 |
| GO:0005178~integrin binding | 2.0E-04 | LAMA5, ITGA2, LAMA3, IGF2, PTN, NPNT, FGF1, L1CAM, FRMD5, IBSP, PTPRZ1, TSPAN8, TNN, ITGA10, ITGA8, ITGB8, ITGBL1, DMD | 4 |
| GO:0007417~central nervous system development | 2.8E-02 | BCAN, ARNT2, ACAN, SCIN, COL2A1, PAX8, GABRA4, SPINK5, NRCAM, SOX6, HAPLN4, HAPLN1 | 4 |
| GO:0030334~regulation of cell migration | 3.5E-02 | LAMA5, FRMD5, LAMA2, TNN, LAMA4, LAMA3, SPATA13, SDC3, PLXNB1, PITX2, PLXNA4, EPHA3 | 4 |
| GO:0008083~growth factor activity | 1.8E-02 | MIA, IGF2, BMP8A, VEGFC, INHBB, INHBA, PTN, NRG2, FGF1, BMP6, FGF14, PDGFC, NRTN, FGF10 | 3 |
| GO:0005576~extracellular region | 2.9E-25 | DMP1, FGF1, IBSP, TNN, IGLON5, LIPG, NRTN, NRCAM, CAR11, KIRREL3, CCBE1, MIA, IGFBP5, WNT5B, SPINK5, NPNT, BCAN, OLFM1, ACE2, SFRP4, OLFM2, COL4A4, SPARCL1, COL8A1, PLA2R1, SCG2, EPHA3, WFDC12, CEMIP, CFH, NXPH4, ENDOD1, PCDH15, DPT, LTBP2, TNFRSF11B, LTBP1, AK7, SCUBE2, SCUBE3, PDGFC, ADAMTS17, CLCA1, FAM180A, WNT4, CTLA2A, IGF2, INHBB, INHBA, SERPINA3N, BMP6, COL1A1, BGLAP2, COL9A1, ITGBL1, CSPG5, COL9A2, FGF10, IGHM, EDA, COL14A1, UCMA, PCSK9, WFDC1, HAPLN4, CHRDL1, HAPLN1, SMPD3, C1QTNF3, SMPD5, ADAMTSL3, TIMP3, ADAMTS6, MMP2, TNFRSF19, NRG2, WNT9A, WNT16, C1QL4, DCN, IL17RB, GREM1, MMP11, TMEFF2, COL2A1, MMP13, FIBIN, MATN1, MATN4, SMPDL3A, MATN3, B4GALT4, LAMA5, MEGF6, SEMA3C, LAMA2, PCOLCE2, DNAH5, COL11A1, SEMA3A, LAMA4, COL11A2, LAMA3, NTN4, PRELP, PTN, SEMA3F, THBS2, ACAN, IL1RL1, CCL8, PTPRZ1, BGLAP, CHADL, EPYC, GPC2, SLIT3, CILP2, TUB, LUM, LGI4, BMP8A, VEGFC, PRSS35, NYX, PLXDC1, POMC, SMOC2, ADM2, MGAT4A, MFAP2, TLL1, CCDC3, FMOD, SCRG1 | 3 |
| GO:0007275~multicellular organism development | 1.1E-06 | EDA, RAX, SCX, FGF1, CHRDL1, GLI2, SMPD3, CDH3, MECOM, SIX2, INSC, EPHB2, PITX2, SOX6, DACT1, CCBE1, EPHA7, WNT5B, MSX2, SPINK5, SHROOM2, TET1, WNT9A, WNT16, NPNT, MLF1, ENAH, SFRP4, OLFM1, ISL2, TMEFF2, GAP43, PAX8, CATSPERD, ITGA8, CDH13, HMX2, SEMA3C, AMIGO1, SEMA3A, FOXG1, GREB1L, IGSF9, ARHGAP22, SCUBE2, ZFP57, PDPN, PDGFC, SLIT3, WNT4, FZD6, BMP8A, VEGFC, L1CAM, BMP6, TBX3, HOPX, TBX2, TTLL7, KIF26B, CSPG5, ACKR3, TLL1, FGFR4, HOXD9, FGFR3 | 2 |
| GO:0005509~calcium ion binding | 1.3E-04 | MEGF6, PCDH15, GUCA1A, LTBP2, THBS2, LTBP1, SLC8A1, RASGRP3, SPTA1, SCUBE2, SCUBE3, ACAN, CDH3, BGLAP, REPS2, SLIT3, PCDH1, SLC25A23, RAB11FIP4, TPD52, SYT5, UNC13C, CCBE1, CUBN, PLA2G4F, GALNT3, PCDHB13, NPNT, DLK1, GRIN1, AIF1L, BCAN, SMOC2, MMP13, SYT12, BGLAP2, MATN1, HRC, CDH13, SPARCL1, ALPL, TLL1, FAT4, MATN4, MATN3 | 2 |
| GO:0005615~extracellular space | 3.6E-10 | XYLT1, FGF1, IBSP, TNN, DPYSL3, LIPG, CCBE1, IGFBP5, WNT5B, BCAN, OLFM1, ACE2, SFRP4, PROCR, OLFM2, COL4A4, COL4A6, SPARCL1, COL8A1, NPTXR, SCG2, WFDC12, CFH, PCDH15, TNFRSF11B, SCUBE2, SCUBE3, PDGFC, WNT4, LRRN3, IGF2, INHBB, INHBA, SERPINA3N, BMP6, COL1A1, RAET1D, VNN1, FAM151A, BGLAP2, COL9A1, ALPL, COL9A3, COL9A2, ADA, FGF10, IGHM, EDA, COL14A1, PCSK9, WFDC1, HAPLN4, HAPLN1, MMP23, C1QTNF3, TIMP3, EAR2, ADAMTS9, PTGIS, MMP2, NRG2, WNT9A, WNT16, C1QL4, DCN, GREM1, MMP11, COL2A1, MMP13, MMP15, CDH13, SMPDL3A, LAMA5, HPGD, SEMA3C, COL11A1, SEMA3A, COL11A2, PRELP, PTN, SEMA3F, ACAN, IL1RL1, CCL8, PTPRZ1, BGLAP, CHADL, SLIT3, CILP2, CKB, LINGO1, LUM, LGI4, CMTM5, BMP8A, VEGFC, NYX, DLK1, POMC, SMOC2, FABP3, TLL1, FMOD, RAMP1 | 2 |
| GO:0030154~cell differentiation | 1.9E-03 | EDA, THRB, SCX, FGF1, CHRDL1, GLI2, FOXQ1, NHSL2, NHSL1, MECOM, PEG10, INSC, SOX6, NKX3-2, SOX5, OSR2, SPINK5, NPNT, CBFA2T3, MLF1, ENAH, ARMC2, SFRP4, TMEFF2, GAP43, PAX8, CATSPERD, ITGA8, HMX2, MYRF, SEMA3C, ARHGEF28, AMIGO1, SEMA3A, TCF7, CPLX2, IGSF9, ARHGAP22, SLIT3, WNT4, CAV3, CCDC136, BMP8A, VEGFC, L1CAM, BMP6, DLK1, HOPX, TTLL7, CSPG5, TLL1, FGF10 | 2 |

**Table S2.** GO Term DAVID analysis of genes up regulated in MC vs KASH expressing MSCs. All presented genes were p<0.05 between treatment groups and had a Log Fold Change >1

| Term | Benjamini | Genes | Fold Enrichment |
| --- | --- | --- | --- |
| GO:1903307~positive regulation of regulated secretory pathway | 1.0E-02 | RAB3A, RAB15, RAB3D, RAB27A, UNC13D | 19 |
| GO:0004028~3-chloroallyl aldehyde dehydrogenase activity | 1.4E-02 | ALDH3A1, ALDH3B2, ALDH1A3, ALDH3B1, ALDH1A1, ALDH1A7 | 11 |
| GO:0010875~positive regulation of cholesterol efflux | 5.0E-05 | ABCA1, ABCA8A, CAV1, ADIPOQ, NR1H3, NFKBIA, CES1A, CES1D, CES1F, CES1G, PPARG, APOE, ABCG1 | 8 |
| GO:0005044~scavenger receptor activity | 4.3E-04 | COLEC12, ENDOU, CD163, MEGF10, STAB1, ENPP2, ACKR3, ENPP1, PRG4, CD36, CXCL16 | 7 |
| GO:0017147~Wnt-protein binding | 4.4E-03 | SFRP1, FZD2, SFRP2, FRZB, FZD7, FZD9, LRP5, NID1, APCDD1, TRABD2B | 6 |
| GO:0017147~Wnt-protein binding | 4.4E-03 | SFRP1, FZD2, SFRP2, FRZB, FZD7, FZD9, LRP5, NID1, APCDD1, TRABD2B | 6 |
| GO:0004364~glutathione transferase activity | 3.7E-02 | GSTM4, GSTK1, GSTM2, GSTA4, MGST3, GSTT3, MGST1, MGST2, GSTT1 | 5 |
| GO:0007156~homophilic cell adhesion via plasma membrane adhesion molecules | 3.2E-09 | RET, PCDHGB8, PCDHGB7, PCDHGB6, PCDHGB5, PCDHGB4, CLSTN3, PCDHGB2, PCDHB22, PCDHB21, PRTG, PCDHA4, EMB, PCDHGA7, PCDHGA6, PCDHGA5, PCDHGA4, PCDHGC5, PCDHGC4, PCDHGA2, PCDHGA1, PCDHB15, PCDHB14, PCDHB13, PCDHB12, PCDHB11, PCDHB10, PCDHGA9, PCDHGA10, PCDHGA12, DCHS2, PCDHB16, PCDHGB1, PCDHB4, PCDHB9, PCDHB8 | 4 |
| GO:0042632~cholesterol homeostasis | 2.7E-03 | ABCA1, ERRFI1, DGAT2, CAV1, EPHX2, LRP5, PCSK9, NR1H3, LPL, CYP39A1, CES1A, FABP4, CES1D, CES1F, CES1G, APOE, SCD1, APOB, ABCG1 | 4 |
| GO:0031012~extracellular matrix | 6.9E-09 | FBLN7, SNED1, LRRC15, TNXB, LAMA1, FBLN1, THBS2, FGF1, NID1, ADAMTS12, OLFML2A, ADAMTS4, CHAD, ADAMTSL2, TNR, EMILIN1, SVEP1, APOE, TIMP4, ADAMTS7, CCBE1, EGFL6, AHSG, TGFB3, RARRES2, MMP3, RTN4RL2, NAV2, KAZALD1, ASPN, MUC5AC, MMP11, MMP14, MMP13, MMP15, CRISPLD2, PXDN, TGFBI, LGR6, MATN4, MATN3 | 3 |
| GO:0030198~extracellular matrix organization | 1.1E-04 | TNXB, FBLN1, NID1, ADAMTS12, OLFML2A, ADAMTS4, ADAMTSL2, TNR, EMILIN1, SOX9, ADAMTS7, PDGFRA, EGFL6, APLP1, MMP3, KAZALD1, AGT, MMP11, MMP14, MMP13, MMP15, CRISPLD2, SMOC1, PXDN, COL4A6, COL8A2, TGFBI, MATN4, MATN3 | 3 |
| GO:0005811~lipid particle | 4.7E-03 | DGAT2, CLSTN3, CAV1, AQP7, ABHD5, CIDEC, DHRS3, ALDH3B2, CES1A, LIPE, CES1D, CES1F, CES1G, PLIN4, PLIN1, APOB, PNPLA2, ADIG | 3 |
| GO:0007155~cell adhesion | 1.9E-13 | RET, SLC23A2, PCDHGB7, PCDHGB6, PCDHGB5, MEGF10, ITGB4, PCDHGB4, CLSTN3, ITGB3, PCDHGB2, ITGAE, SIGLECG, COMP, STAB1, CYP1B1, EMILIN2, TNR, SVEP1, EMILIN1, EMB, CD36, CD177, PCDHGA7, PCDHGA6, EGFL6, PCDHGA5, CXADR, PCDHGA4, CD93, PCDHGA2, PCDHGA1, APLP1, PCDHGA9, CLDN15, ITGAD, PXDN, ADAM12, PCDHGB1, COL8A2, IGFALS, TLN2, NLGN3, FBLN7, TNXB, LAMA1, LAMA3, NEDD9, DPT, NID1, THBS2, PCDHB22, KLC1, LY9, PCDHB21, SRPX2, PCDHA4, CTNNAL1, COL28A1, AOC3, VCAM1, LAMB3, JUP, RNASE10, PCDHGC5, PCDHGC4, PCDHB15, PCDHB14, PCDHB13, PCDHB12, BMX, PCDHB11, SULF1, PCDHB10, PCDHGA10, PCDHGA12, FAP, KITL, FES, PCDHB16, PMP22, ACKR3, PCDHB4, SIGLEC1, TGFBI, PCDHB9, PCDHB8 | 3 |
| GO:0006935~chemotaxis | 4.7E-02 | PDGFRA, C5AR2, RARRES2, CXCR5, CXCL1, PIK3CG, CXCL16, CCL9, CCL8, CXCL12, FES, CCL6, KIT, ENPP2, CCL2, ACKR3, EAR2, PF4, CMKLR1 | 3 |
| GO:0008017~microtubule binding | 4.2E-03 | MACF1, CCDC69, MAST1, GLI1, BCL2L11, NUF2, NUSAP1, CHP1, KIF1A, FSD1, FAM83D, APC2, MX2, PLK1, STARD9, MTUS1, MTUS2, KIF24, EML1, EML2, DCLK1, CAMSAP3, DNM3, CENPE, CENPF, KIF26A, FES, MAP1A, CCDC88C, CCDC170, TRIM54, CRYAB, SPC24 | 2 |
| GO:0005509~calcium ion binding | 1.8E-06 | RET, PCDHGB8, SNED1, PCDHGB7, PCDHGB6, PCDHGB5, PCDHGB4, CLSTN3, PCDHGB2, COMP, THBD, CAPNS2, STAB1, ENPP2, ENPP1, CHP1, SVEP1, ENPP3, PCDHGA7, CCBE1, PCDHGA6, EGFL6, PCDHGA5, PCDHGA4, CD93, ACTN3, HEG1, PCDHGA2, PCDHGA1, ASPN, PCDHGA9, MAN1A, MMP13, PCDHGB1, CASQ2, S100A5, S100A4, GAS6, MATN4, MATN3, FBLN7, MACF1, LPL, FBLN1, NID1, THBS2, RASGRP2, PCDHB22, PCDHB21, NKD2, MAN1C1, PCDHA4, AOC3, S100G, GSN, PCDHGC5, PCDHGC4, SUSD1, PCDHB15, PCDHB14, PCDHB13, PCDHB12, SYT13, PCDHB11, SULF1, PCDHB10, GRIN1, SULF2, PCDHGA10, EHD2, PCDHGA12, CABYR, DCHS2, SMOC1, PCDHB16, CD248, PCDHB4, MEGF8, PCDHB9, PCDHB8 | 2 |
| GO:0003779~actin binding | 3.7E-02 | FHOD3, MACF1, ANKRD35, FAM107A, DIXDC1, SLC6A2, MYLK, PDLIM1, ABLIM2, OPHN1, STK38L, EPS8L1, TNNI3, PHACTR1, VILL, TNS4, SYNPO, TNS1, MYH7B, MARCKSL1, GSN, ACTN3, MYO10, ANG2, SHROOM3, KLHL3, VASH1, SPTB, CORO2B, MTSS1, SYN1, SMTN, DAAM2, TNNT2, MAP1A, SPIRE2, ANG, S100A4, TLN2 | 2 |
| GO:0005576~extracellular region | 5.6E-13 | IL1RN, SERPINE2, PMCH, HP, F13A1, PLAT, FGF1, OLFML2A, IFI30, CXCL14, CLU, CNDP1, COMP, C4B, ENDOU, CAR15, C1RL, CCL27A, ENPP2, ENPP1, TNR, SVEP1, ENPP3, PRL2C2, IL15RA, CCBE1, WFDC21, ST6GAL1, CXADR, ADIPOQ, RNASE4, LIFR, VASH1, BTC, OLFM1, SFRP1, SFRP2, CRISPLD2, COL8A2, SERPING1, S100A4, IGFALS, PLA2R1, SCG2, PRL2B1, CFD, FBLN7, PTGFR, CEMIP, HTRA3, LPL, DPT, FBLN1, NID1, ADAMTS12, KNG2, KNG1, C3, SERPINA3C, CHAD, FAM180A, APOD, WFDC18, APOE, APOB, COL28A1, CD163, VCAM1, CDSN, RNASE10, TGFB3, RARRES2, TFPI2, TNFSF13, PRG4, SERPINA3N, BMP6, BMP5, ABHD15, IL1F10, CXCL12, TMEM98, CILP, KITL, FGF18, LCN2, ITGBL1, TGFBI, DRAXIN, SNED1, ORM1, PZP, PCSK9, TRF, ADM, CXCL1, PCSK6, CHRDL1, WFDC3, ADAMTS4, SMPD3, ESM1, C1QTNF1, BC028528, C1QTNF6, ADAMTSL2, EMILIN2, EMILIN1, CPXM1, LBP, INSL3, TIMP4, CAMP, ADAMTS7, EGFL6, AHSG, TNFRSF18, ANG2, MMP3, NRG1, C1QL4, OTOR, ASPN, IL17RB, GFRA4, TGFBR3, MMP11, MMP13, PXDN, ANG, GAS6, MATN4, CRYAB, MATN3, B4GALT4, TNXB, LAMA1, DNAH5, SAA3, COL11A2, LAMA3, NTN4, SEMA3G, FURIN, RETN, THBS2, CST3, NTF5, SERPINB1A, CCL9, CCL8, SRPX2, CCL6, FRZB, PATE2, CCL2, CILP2, GSN, ANGPT1, LAMB3, GDF15, IL34, APOC4, PRSS35, SULF1, PLXDC1, KAZALD1, SULF2, FAP, SMOC1, APOC1, SIGLEC1, CCDC3, INHA, PF4 | 2 |
| GO:0007049~cell cycle | 2.7E-02 | ERRFI1, ERCC6L, NCAPG2, FAM107A, BUB1B, BRCA1, MKI67, SMPD3, RGS2, CHAF1B, RASSF2, MIS18BP1, NUF2, NUSAP1, WDR6, OIP5, CEP55, LIG1, MTUS1, ESCO2, VASH1, KNSTRN, ASPM, TXNIP, MCM4, GAS1, MCM5, CCNO, ITGB3BP, LLGL2, CDCA3, CDCA5, TXLNG, NEDD9, AURKB, NCAPH, BRINP1, BCL2L11, CLSPN, FSD1, FAM83D, BUB1, E2F7, E2F8, PLK1, INCA1, CABLES1, FOXN3, TICRR, CENPE, RGCC, NR4A3, CCNG2, PMP22, SPC24, SPC25, CDKN3 | 2 |
| GO:0005615~extracellular space | 1.1E-07 | IL1RN, APOL9B, SERPINE2, PMCH, HP, XYLT1, PLAT, FGF1, OLFML2A, CXCL14, CLU, CXCL16, COMP, LGALS4, C4B, LIPE, C1RL, CCL27A, ENPP2, ENPP1, TNR, SVEP1, PRL2C2, CCBE1, WFDC21, ENTPD1, CXADR, ADIPOQ, RNASE4, VASH1, BTC, OLFM1, SFRP1, SFRP2, CRISPLD2, COL4A6, COL8A2, DPEP1, SERPING1, S100A4, SCG2, PRL2B1, H2-D1, CFD, FBLN7, CPB1, LPL, FBLN1, AGER, KNG2, KNG1, C3, SERPINA3C, SERPINA3F, CHAD, APOD, WFDC18, APOE, APOB, COL28A1, VCAM1, TGFB3, RARRES2, TFPI2, TNFSF13, PRG4, SERPINA3N, BMP6, BMP5, IL1F10, CXCL12, TMEM98, PPP1R1A, VNN3, CILP, KITL, FGF18, LCN2, TGFBI, ULBP1, ORM1, PZP, PCSK9, TRF, ADM, CXCL1, GPT, PCSK6, AREG, WFDC3, ADAMTS4, THBD, C1QTNF1, C1QTNF6, EMILIN1, CPXM1, LBP, EAR2, INSL3, TIMP4, CAMP, AHSG, ANG2, MMP3, NRG1, RTN4RL2, C1QL4, ASPN, MUC5AC, GFRA4, TGFBR3, MMP11, MMP14, MMP13, MMP15, PXDN, KIT, ANG, GAS6, LRRC15, TNXB, LAMA1, SAA3, COL11A2, H2-Q7, MILL2, RETN, CST3, NTF5, SERPINB1A, CCL9, CCL8, SRPX2, CCL6, FRZB, OLFML3, PATE2, CCL2, CILP2, CKB, AOC3, S100G, CAR2, GSN, ANGPT1, GDF15, IL34, SULF1, KAZALD1, AGT, SULF2, KRT18, LRG1, FAP, SMOC1, LGR6, INHA, RAMP1, PF4 | 2 |
| GO:0005886~plasma membrane | 3.2E-04 | LY6C2, SLC23A2, ERRFI1, HDAC11, MRGPRF, AQP7, MRGPRE, SLC4A4, MYLK, LY6C1, RXFP3, LIPE, RASSF2, CAPNS2, PIEZO2, SYNPO, EPHB3, PDK1, CMKLR1, TMEM88B, IL15RA, ENTPD1, ENTPD2, PAQR7, CXADR, ACTN3, DAPK1, SLC11A1, MTUS1, SLC6A12, UNC5C, SYTL5, KNSTRN, SYTL3, BTC, AR, SFRP1, ADCY9, FNDC1, TFR2, TLN2, H2-D1, LLGL2, SLC22A4, MACF1, PTGFR, ADCYAP1R1, TTYH3, NLRX1, STXBP2, MGST2, APCDD1, PPL, SLC5A3, LY9, ADH1, MC2R, GALR2, PRKAR2B, PCDHA4, ATP6V0A4, PIP5K1B, APOE, TRPM6, RALGDS, ABCA1, BBS1, FZD2, MAGEE1, DMPK, JUP, FZD7, ABCA4, PDE2A, FZD9, PCDHGA10, EHD2, SLC6A6, PCDHGA12, SLC6A7, VANGL2, TMEM98, VNN3, KITL, DCHS2, NOX4, PCDHB4, GRAP, CPEB3, RGL1, PCDHB9, PCDHB8, XKR5, ACVRL1, RET, CLIC5, A530016L24RIK, PTPRN, GRIK5, PCSK9, PTPRJ, TMEM182, BRCA1, MS4A4D, SLC6A2, SIGLECG, CHRDL1, SMPD3, SMPD2, TUBA1A, GNGT2, C1QTNF1, STAB1, FFAR2, CD36, EMB, GPR135, LYNX1, SLC2A10, HEG1, AVPR1A, SYN2, IL17RB, ANO3, GFRA4, RGMA, RNF144A, MRAP, SLC7A4, GPRC5B, MMP14, ADRB3, CLDN15, SLCO4A1, CDC42EP5, KIT, PLIN4, CDC42EP2, GAS1, TLR8, HCLS1, SGK1, CRYAB, VAMP5, RAPGEF3, VAMP2, RAPSN, LRRC15, SLC43A2, PTGER3, ADCY4, CXCR5, NEDD9, PCDHB22, PCDHB21, ADCY7, NKD2, CST3, REM1, GLRA1, GPR153, HAS2, SLC17A7, CKB, SLC17A1, JAM2, MPZL1, GPR157, AOC3, CAR2, MPP2, GSN, SORT1, RFTN1, PCDHB15, PCDHB14, PCDHB13, PCDHB12, SULF1, PCDHB11, PLXDC1, PCDHB10, SULF2, GRIN1, TJP1, KRT19, FAP, STAC2, MC5R, SIGLEC1, LGR6, RAMP1, LGR5, PNPLA2, SEMA5A, KLB, RAB3A, GPR68, CLSTN3, RAB3D, TRAC, FAM107A, RIMS1, RGS2, GRB14, SLCO2B1, CAR15, ENPP2, CHP1, ENPP1, CAR14, ENPP3, CD177, EPM2A, PDGFRA, SLC15A2, CD93, ABCA8A, RALGAPA2, APLP1, LIFR, CYS1, KCTD7, GPRIN3, DISP2, EDAR, HIC2, ALDH3A1, CACNB3, SCN8A, ADAM12, DPEP1, PLA2R1, ABCG1, PDE9A, LY6F, TRABD2B, RTN4R, RTN2, CEMIP, NDRG4, NPR1, C5AR2, NPR3, GPR88, ARHGAP19, LPL, ARHGAP18, CSF2RB, CACNA1C, RASGRP2, EFNA4, AGER, CACNA1G, HCAR2, HCAR1, ALDH3B1, CD300LB, TNFRSF14, SLC38A2, ASIC1, SLC38A4, MARK1, GGT5, SLC14A1, OSBPL7, CD163, VCAM1, RASL10B, CAV1, ARHGEF39, TRPV3, TRPV1, BMX, POU2F3, NFKBIA, DAB2, EFNA2, FES, TMEM119, SPIRE2, SLC26A7, PLXNB1, SLC26A4, ULBP1, GPSM1, PCDHGB8, SNAP25, DOCK5, KCNK6, PCDHGB7, PCDHGB6, PCDHGB5, MEGF10, PCDHGB4, ITGB4, ITGB3, DOCK8, PCDHGB2, MAST1, SLC40A1, TRF, HSPB1, ARRB1, SLC2A4, TSPAN12, PIK3CG, RERG, THBD, EXO1, WDR6, GM8369, GPSM3, PCDHGA7, PCDHGA6, PCDHGA5, SLC36A2, PCDHGA4, KSR1, PCDHGA2, PDE4D, ABCC5, TNFRSF18, PCDHGA1, SH2D3C, SHROOM3, NRG1, RTN4RL2, SSTR3, SPTB, PCDHGA9, DNM3, TGFBR3, SLCO1C1, ADORA2A, RTN4RL1, PCDHGB1, TNFRSF25, NLGN3, RGS13, H2-Q7, LRP5, SEMA3G, NTN4, MILL2, FURIN, ATP1A2, ASAP2, SAMHD1, PRTG, TGFBR3L, CNNM1, CLMP, ACVR1C, CNR1, EPS8L1, S1PR3, CTNNAL1, CYTH1, MARCKSL1, ANGPT1, MYO10, PCDHGC5, PCDHGC4, STYK1, KCNJ15, ATP2B4, SYT13, TSHR, P2RX7, RAB15, PMP22, ACKR3, CD248, PIK3AP1, CDO1, KCNK3 | 1 |
